# Supplementary material for: Mechanisms underlying TARP modulation of the GluA1/2-γ8 AMPA receptor
Source: Nat Commun. 2022 Feb 8;13:734. doi: 10.1038/s41467-022-28404-7 (PMC8826358; doi:10.1038/s41467-022-28404-7)
Supplement: Supplementary file 1 — Supplementary Information [file 41467_2022_28404_MOESM1_ESM.pdf]

Supplementary Information for

Mechanisms underlying TARP modulation of the GluA1/2-γ8 AMPA receptor

Beatriz Herguedas[§] , Bianka K. Kohegyi[§], Jan-Niklas Dohrke, Jake F. Watson, Danyang Zhang, Hinze Ho, Saher A. Shaikh, Remigijus Lape, James M. Krieger and Ingo H. Greger[*]

[§] these authors contributed equally; * for correspondence: ig@mrc-lmb.cam.ac.uk

Containing:
Supplementary Figures 1-12
Supplementary Tables 1 and 2

Supplementary Figures

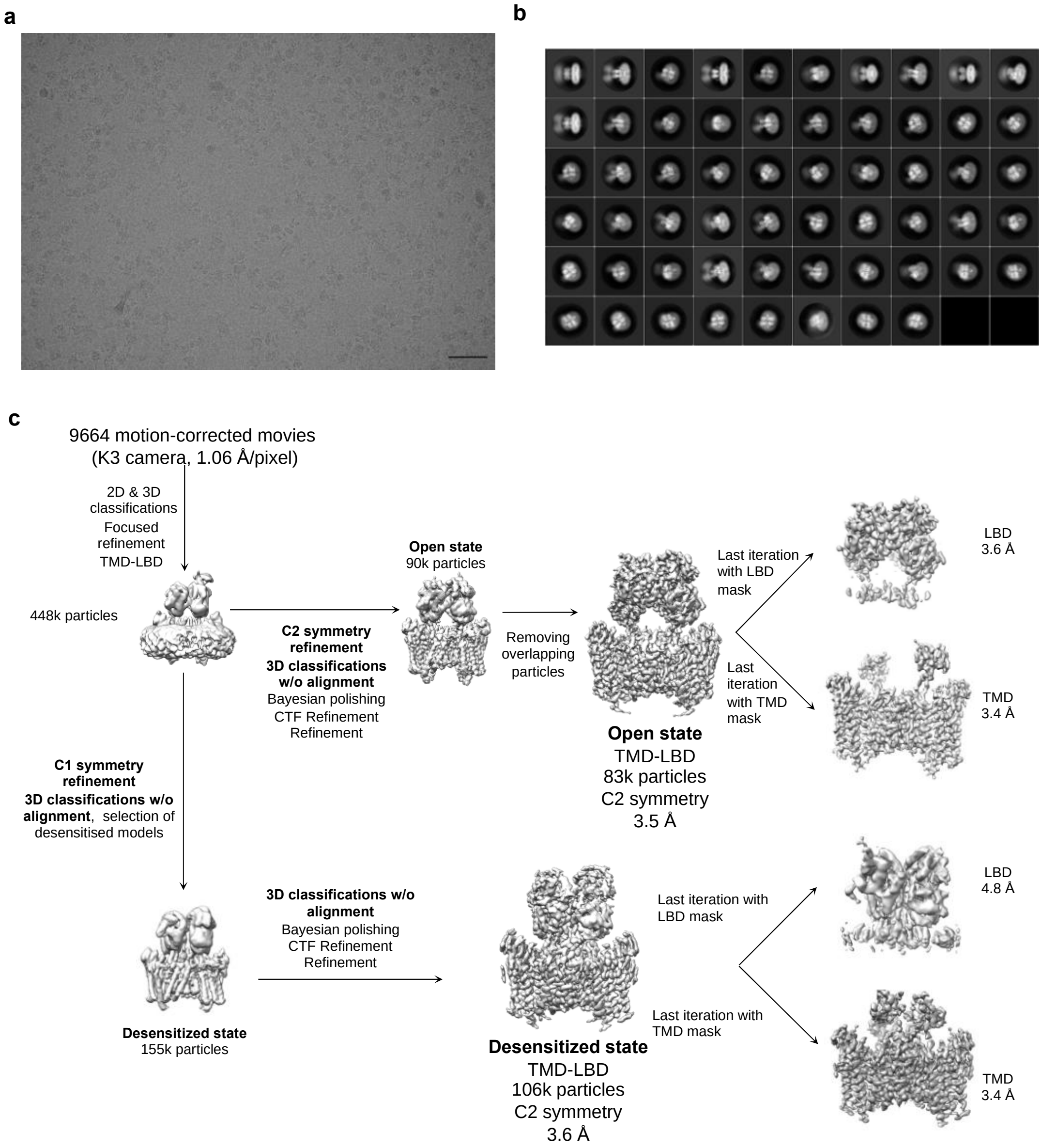

Supplementary Fig.1: Cryo-EM data collection and processing for GluA1/2 TARP-γ8 open and desensitized states.

a, Representative motion-corrected micrograph collected on an FEI Titan Krios with a K3 detector; scale bar 50 nm; 9664 micrographs were collected. **b,** 2D averages (box 320 px) of open and desensitized particles **c**, Workflow of cryo-EM data processing. After motion correction and 2D and 3D classification to remove non-AMPAR particles with binned particles, a subset of 448,038 was subjected to 3D refinement applying either C1 symmetry or C2 symmetry. 3D refinement in C1 symmetry followed by 3D classification without alignment resulted in a subclass with desensitized features. Using 3D classification w/o alignment, followed by Bayesian polishing, CTF Refinement and 3D refinement, a final desensitized map was obtained, with an overall resolution of 3.6 Å after postprocessing. Refinement of the 448,038 particles in C2 symmetry followed by 3D classification w/o alignment resulted in a subclass with open-state features. Bayesian polishing and CTF refinement further improved the quality of the maps. The particles from open and desensitized state were compared and duplicated particles were used separately for 3D refinement, in order to determine whether they were mainly open or desensitized. As these particles resulted in a desensitized model, they were removed from the original open state subset and another 3D refinement was performed. To improve the quality of the maps in the TMD and LBD layer, masks were generated for TMD and LBD sectors, and the 3D refinement was

continued from the last iteration in RELION applying this soft mask, generating TMD and LBD maps with improved quality.

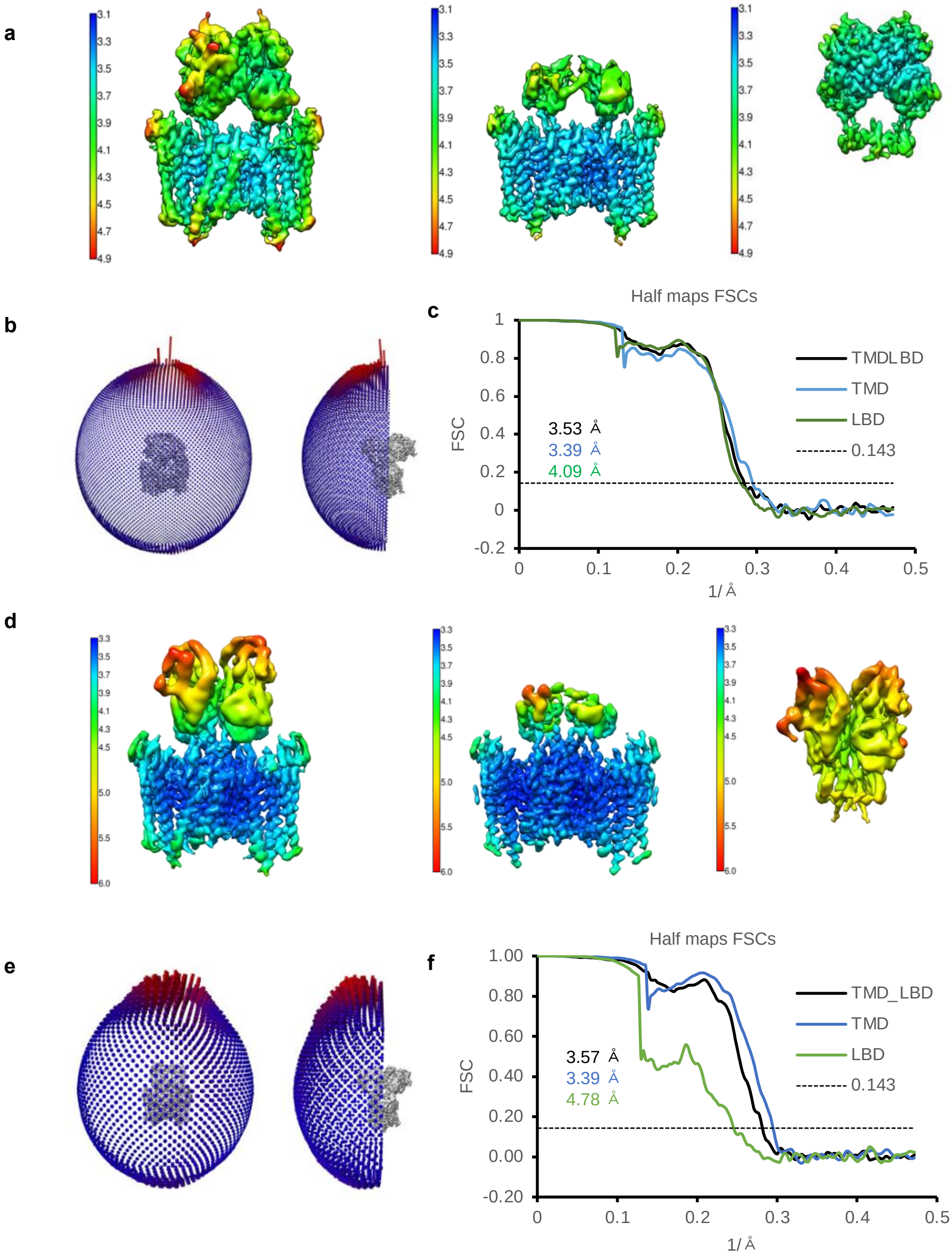

Supplementary Fig. 2: Fourier shell correlations, particle orientations and local resolution of cryo-EM maps.

a, Local resolution of open-state TMD/LBD, TMD and LBD maps. **b,** representation of the particle distribution of the TMD/LBD particles **c,** Fourier Shell Correlation of the half maps plotted as a function of spatial frequencies for TMD/LBD, TMD and LBD open 3D refinements. Resolution estimates based on the FSC=0.143 threshold criteria are indicated. **d,** Local resolution of desensitized state TMD/LBD, TMD and LBD maps. **e,** representation of the particle distribution of the TMD/LBD particles **f,** Fourier Shell Correlation of the half maps plotted as a function of spatial frequencies for TMD/LBD, TMD and LBD desensitized 3D refinements. Resolution estimates based on the FSC=0.143 threshold criteria are indicated.

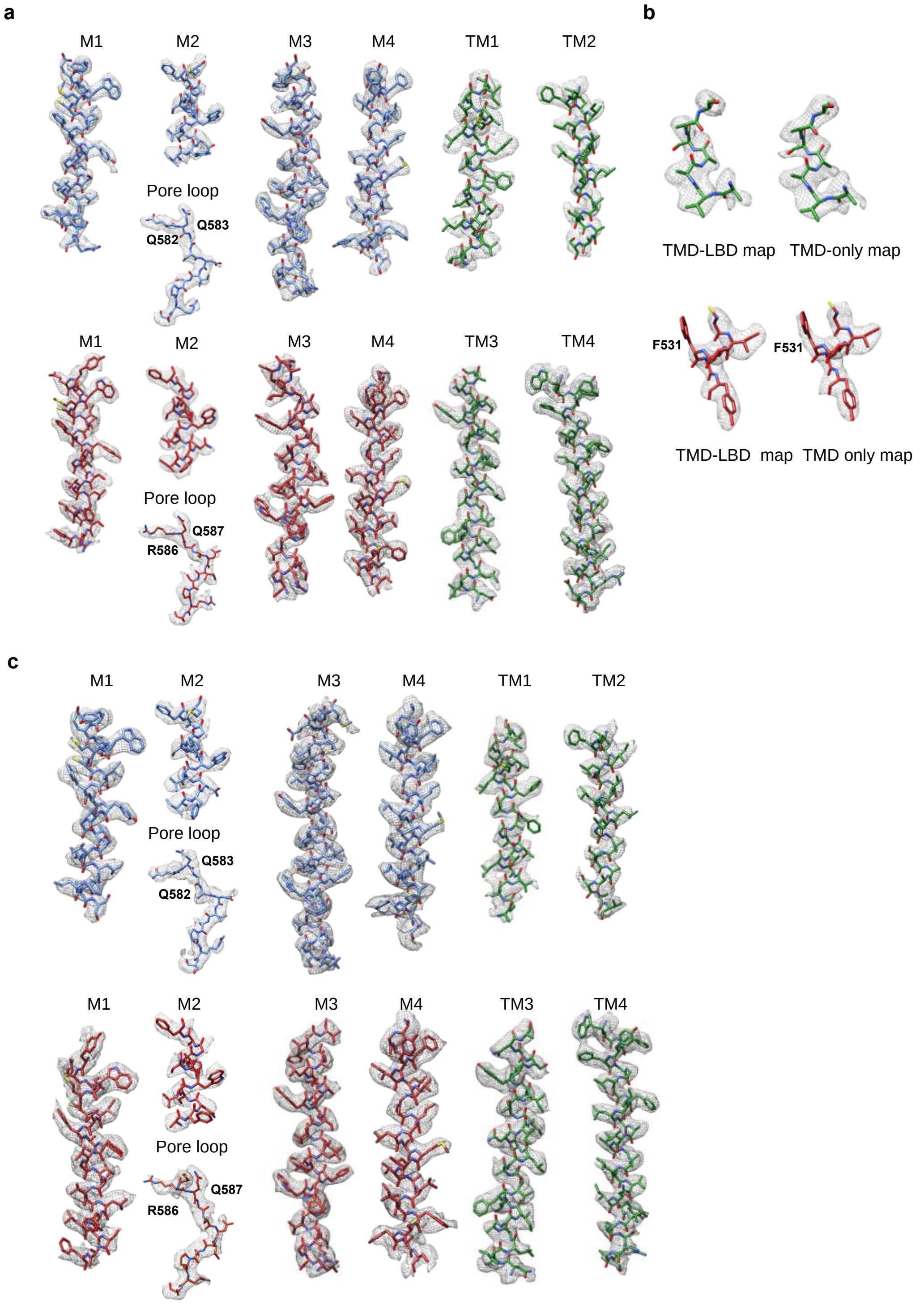

Supplementary Fig. 3: GluA1/2 TARP-γ8 side chain EM-densities of transmembrane and pore loop segments.

a, Details of TMD helices fitting into the cryo-EM open-state TMD map. GluA1, GluA2 and TARP are coloured in blue, green and red, respectively, and main residues of the pore are labelled. **b**, comparison of densities in TMD-LBD maps vs. TMD only maps. Quality of the cryo-EM map greatly improves for some specific residues, aiding manual modelling. **c,** Details of TMD helices fitting into the cryo-EM desensitized-state TMD map. GluA1, GluA2 and TARP are coloured in blue, green and red, respectively.

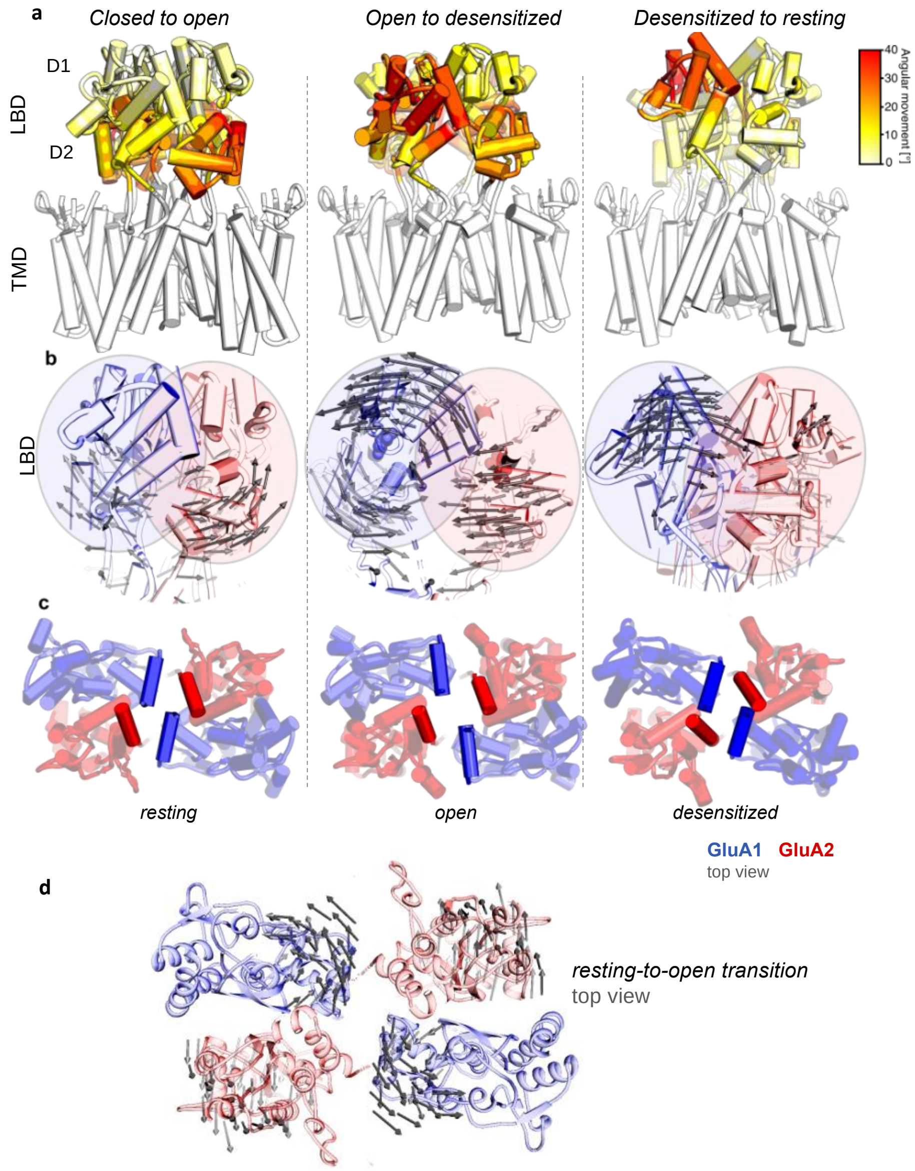

Supplementary Fig. 4: Analysis of LBD layer dynamics during gating transitions.
a, The degree of rotation undergone by the Cα atoms around the centre of rotation of an LBD dimer is shown for each transition by colouring the starting structure from white (least mobile) to red (most mobile): closed to open (left), open to desensitised (middle), desensitised to closed (right). **b**, The vectors connecting the Cα atoms of the same transitions as in **a** are displayed. **c**, A top view of the closed, open and desensitised state shows the differences in the tetrameric LBD conformation. **d**, The conformational change of the whole LBD tetramer is indicated for the closed to open transition in top view.

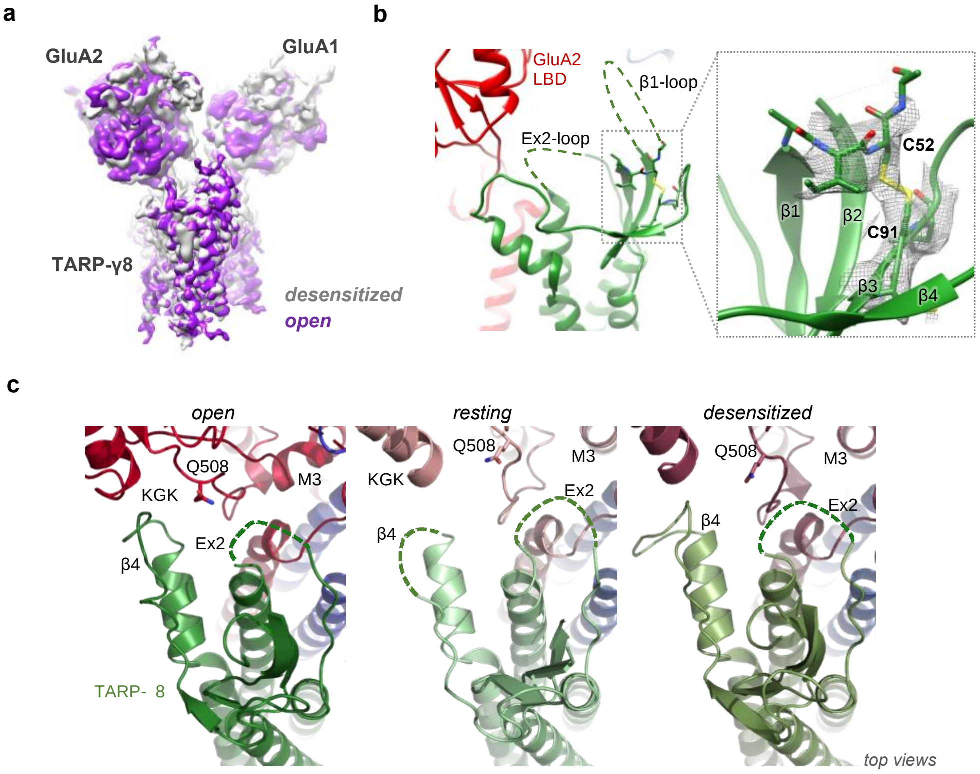

Supplementary Fig. 5: Extracellular TARP-γ8 loop orientation and Cysteine bridge in Ex1.
a, Superposition of desensitized (grey) and open (purple) state maps, with the TARP-γ8 in the front and the two LBDs shown. **b,** Cartoon representation of the TARP-γ8 extracellular region in the open-state complex. Dashed lines denote the residues which are absent in the cryo-EM maps. The zoom on the right shows the cryo-EM density around the C52-C91 disulphide bridge. **c**, Cartoon representation of the TARP-γ8 - GluA2 LBD interacting region in the three functional states, viewed from the extracellular region. Gln508 of GluA2 is represented as sticks, and the main structural elements involved in the interaction are highlighted.

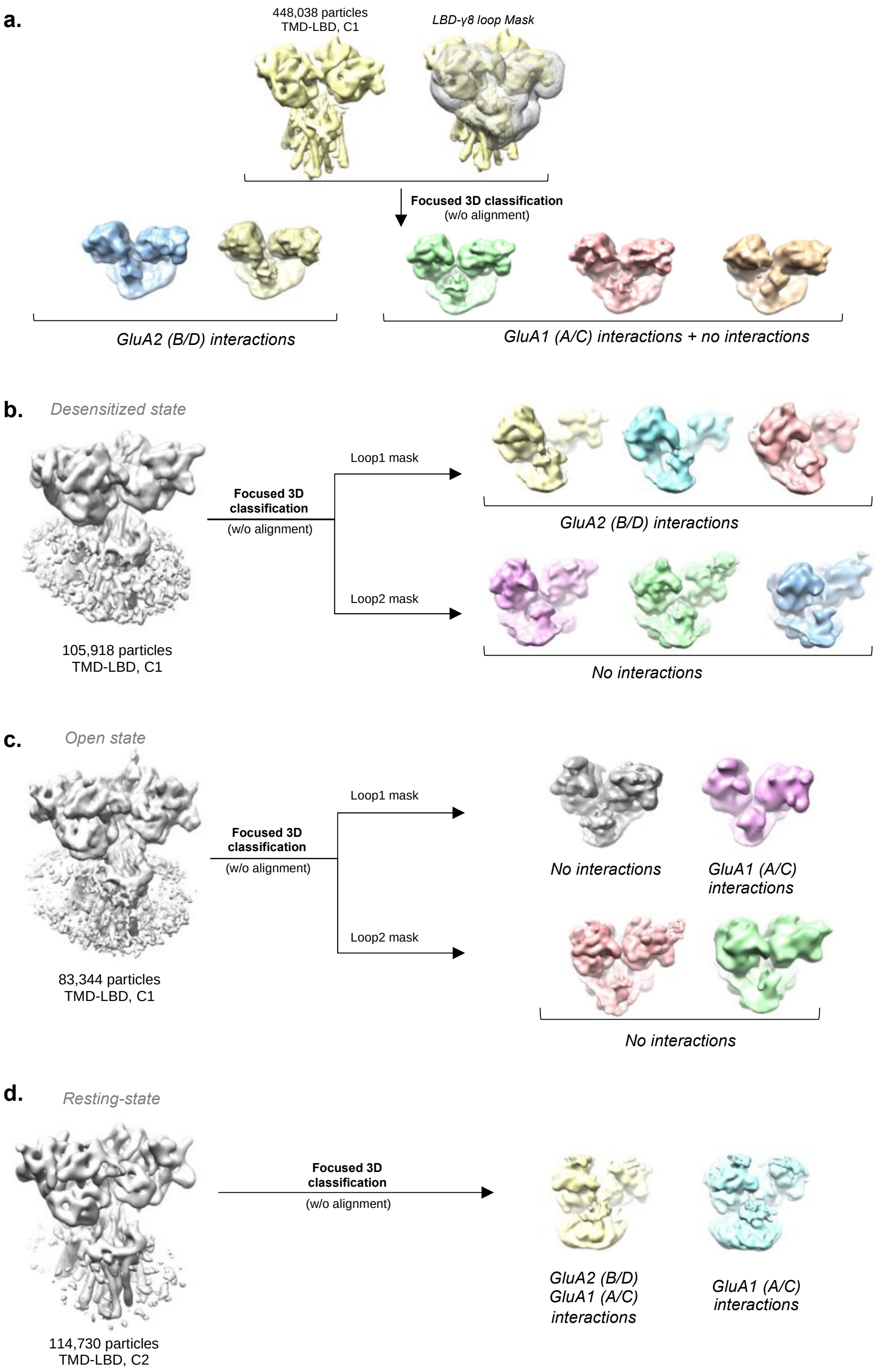

Supplementary Fig. 6: Cryo-EM analysis of the TARP-γ8 extracellular loop interaction with the LBDs.

a) After 3D refinement of the whole dataset (448,037 particles, both open and desensitized, light yellow, C1 symmetry), a mask covering two LBD protomers and the TARP-γ8 extracellular region (ECR) was created (transparent gray). 3D classification without alignment using this mask resulted in five classes: two classes with TARP-γ8 ECR - GluA2 (B/D) LBD interactions (top classification, blue and yellow classes), two classes with TARP-γ8 ECR - GluA1 (A/C) LBD interactions (top classification, salmon and beige classes), and one class with no clear interactions (green class). **b**) Cryo-EM analysis of the TARP-γ8 extracellular loop conformation in the desensitized state particles. After 3D refinement (C1 symmetry, gray map) two masks were generated (as described above) and 3D classification without alignment was performed.

Classifications focused on loop 1 clearly showed TARP-γ8 ECR - GluA2 (B/D) LBD interactions (yellow, cyan and salmon classes) while classifications focused on loop 2 lacked clear interactions (pink, green and blue classes). Two extra classes with a very small number of particles were also obtained (not shown). **c**) Cryo-EM analysis of the TARP-γ8 extracellular loop conformation in the open state particles. After a 3D refinement step (C1 symmetry, gray map) two masks were generated (as described above) and 3D classification without alignment was performed. Classifications focused on loop 1 showed both TARP-γ8 ECR – GluA1 (B/D) LBD interactions (pink class) and no interactions (gray class) while 3D classifications focused on loop 2 lacked clear interactions (salmon and green classes). Three additional classes were obtained with a very small number of particles (not shown). **d**) Cryo-EM analysis of the TARP-γ8 extracellular loop conformation in the resting state particles (used for EMD-4572 reconstruction). TMD-LBD 3D refinement was performed in C2 symmetry as refinement in C1 did not reveal differences between loops. Loop classification revealed a class with both TARP-γ8 ECR – GluA2 (B/D) and TARP-γ8 ECR – GluA1 (A/C) interactions (light yellow class) and one class with TARP-γ8 ECR – GluA1 (A/C) interactions. Three additional classes were obtained with a very small number of particles (classes not shown).

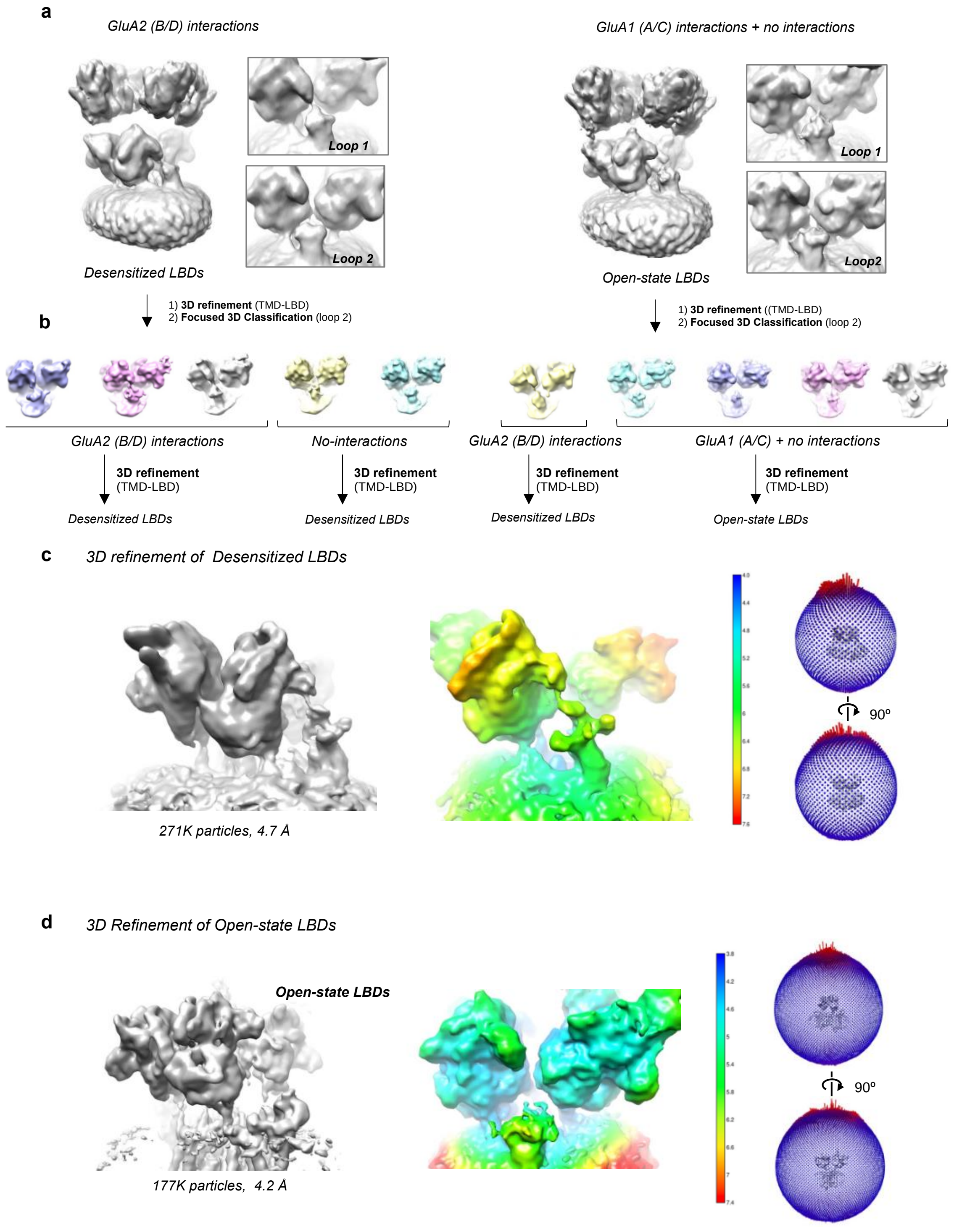

Supplementary Fig. 7: Focused analysis of TARP-γ8 β1 loop interaction with the LBDs.
a, After loop classification of the 448,037 particle-dataset (see Supplementary Fig.6a), classes showing GluA2 (B/D) interactions were pooled together and used for 3D refinement, resulting in a full-length model with desensitized-like LBDs (left panel, full-length model, gray), where one of the loops showed strong signal for GluA2 (B/D)-interactions ("loop 1"), while the other had weaker signal ("loop 2"). Similarly, GluA1 (A/C) classes and classes with no clear interaction were pooled together and refined, resulting in a full-length model with open-like LBDs (right panel, full-length model, gray) and weak or absent GluA1 (A/C) contacts (right panel). **b**, Further 3D classifications focused on the loop with weaker density ("loop 2") are shown below the full-length maps. A class

within the “A/C” pool (light yellow class) had B/D interactions, while two classes within the “B/D pool” had no interactions (light yellow and cyan classes). 3D refinement revealed that both particles in the GluA2 (B/D) pool and particles with mixed-loop features had desensitized LBDs (labelled as “desensitized LBDs”), while the particles with no clear interactions or A/C interactions resulted in reconstructions where the two LBDs were clearly open (labelled as “open-state LBDs”). All the particles with desensitized LBDs were pooled together and used for 3D refinement. **c**, Details of the postprocessed TMD-LBD map, local resolution and particle distribution obtained from refinement of pooled desensitized particles, showing a strong density for the GluA2-loop interaction. **d**, Details of the postprocessed TMD-LBD map, local resolution and particle distribution obtained from pooled “AC+no interaction” particles, which show open-state LBDs.

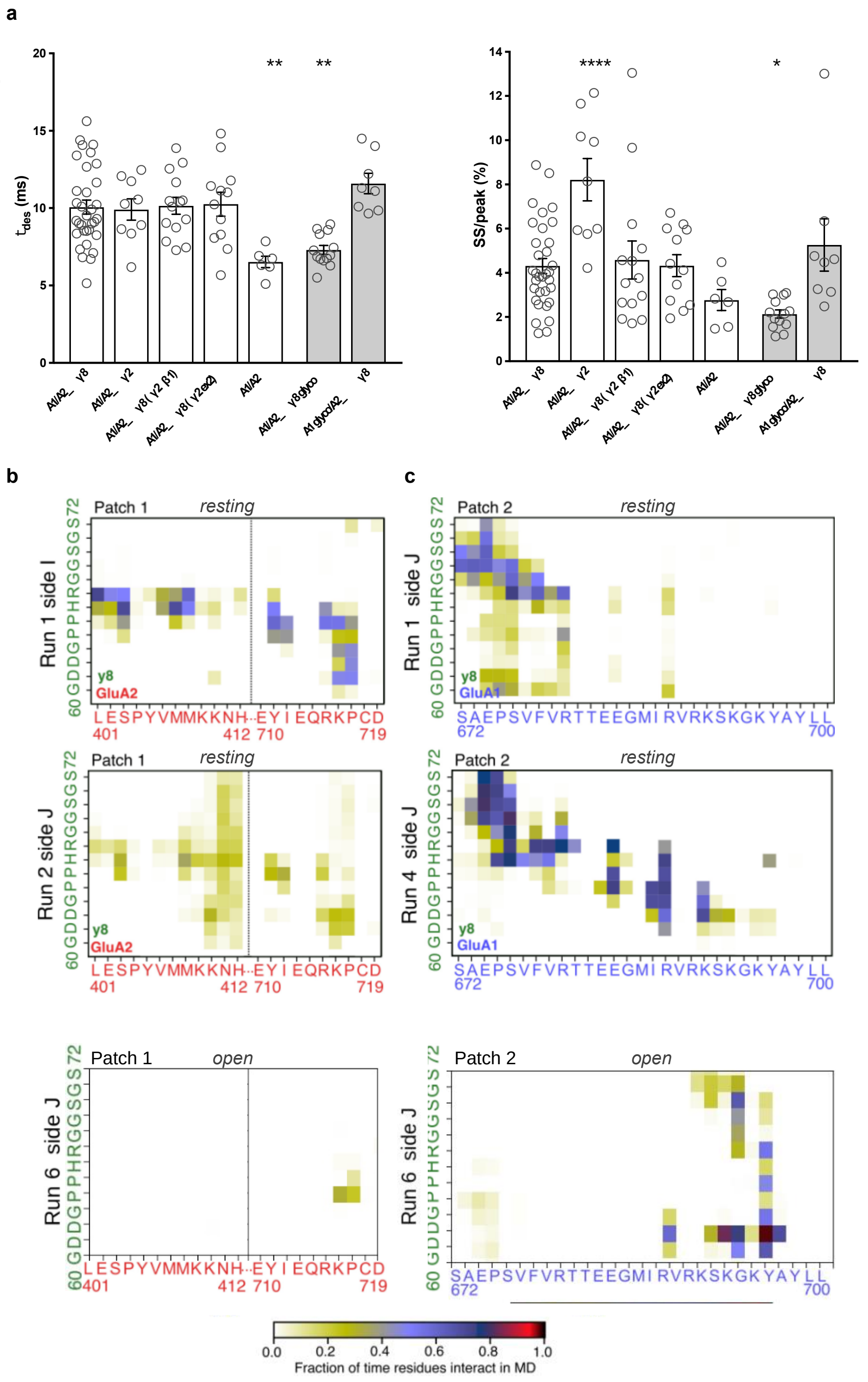

Supplementary Fig. 8: Electrophysiological and MD-simulation analysis of TARP-γ8 β1 loop interaction with the LBDs.

a, Left: Both γ8 and γ2 comparably decelerate the rate of entry into the desensitized state of A1/A2 heteromers, while introducing a glycan at the D1 lobe of GluA2, but not GluA1, functionally decouples the effect of γ8 on desensitization entry rate. A1/A2_γ8, 10.06 ± 0.45 (mean ± SEM), n = 33; A1/A2_γ2, 9.91 ± 0.69, n = 9, (ns, p=0.9998); A1/A2_γ8 (γ2 β1), 10.15 ± 0.55, n = 14, (ns, p=0.9999); A1/A2_γ8 (γ2 ex2), 10.25 ± 0.77, n = 12, (ns, p=0.9997); A1/A2, 6.52 ± 0.37, n = 6,

(**, p=0.0026); A1/A2_γ8 glyco, 7.30 ± 0.29, n = 13 (**, p=0.0013); A1 glyco/A2_γ8, 11.59 ± 0.66, n = 8, (ns, p=0.3677). One-way analysis of variance (ANOVA) with Dunnett's multiple comparison test (against A1/A2_γ8 as control), $p < 0.0001$. Right: A1/A2_γ2 exhibits significantly higher steady-state current compared to A1/A2_γ8, an effect that is not dependent on β1 or the Ex2 loops of γ2. GluA2 D1 glyco mutant displays steady-state current similar to that of TARP-free A1/A2 heteromer. A1/A2_γ8, 4.31 ± 0.34, n = 33; A1/A2_γ2, 8.21 ± 0.96, n = 9, (****, p<0.0001); A1/A2_γ8 (γ2 β1), 4.58 ± 0.86, n = 14, (ns, p=0.9984); A1/A2_γ8 (γ2 ex2), 4.32 ± 0.50, n = 12, (ns, p>0.9999); A1/A2, 2.77 ± 0.48, n = 6, (ns, p=0.5239); A1/A2_γ8 glyco, 2.13 ± 0.19, n = 13, (*, p=0.0234); A1 glyco/A2_γ8, 5.26 ± 1.19, n = 8, (ns, p=0.8436). One-way ANOVA with Dunnett's multiple comparison test (against A1/A2_γ8 as control), $p < 0.0001$. Source data are provided as a Source Data file. **b, c,** Representative heat maps are shown for two regions containing interactions between residues on TARP-γ8 β1 loop (green), and AMPAR GluA1 (blue) and GluA2 (red), called patch 1 (b) and patch 2 (c), respectively. Run1, Run2, Run3 and Run4 were resting state simulations, while Run6 was an open state simulation (see methods for details). Tarp I and J refer to the two TARP-γ8 molecules present in the system, respectively. The heat maps show the frequency of interactions within an inter-atomic cutoff of 5 A (non-H atoms), calculated at 100 ps intervals over the simulation time of 350 ns for each run (2 replicas/system, 6 runs in total). Resting state simulations show interactions of β1 loop with both, patch 1 and patch 2, while in the open state, the β1 loop interacts primarily with patch 2. Source data are provided as a Source Data file.

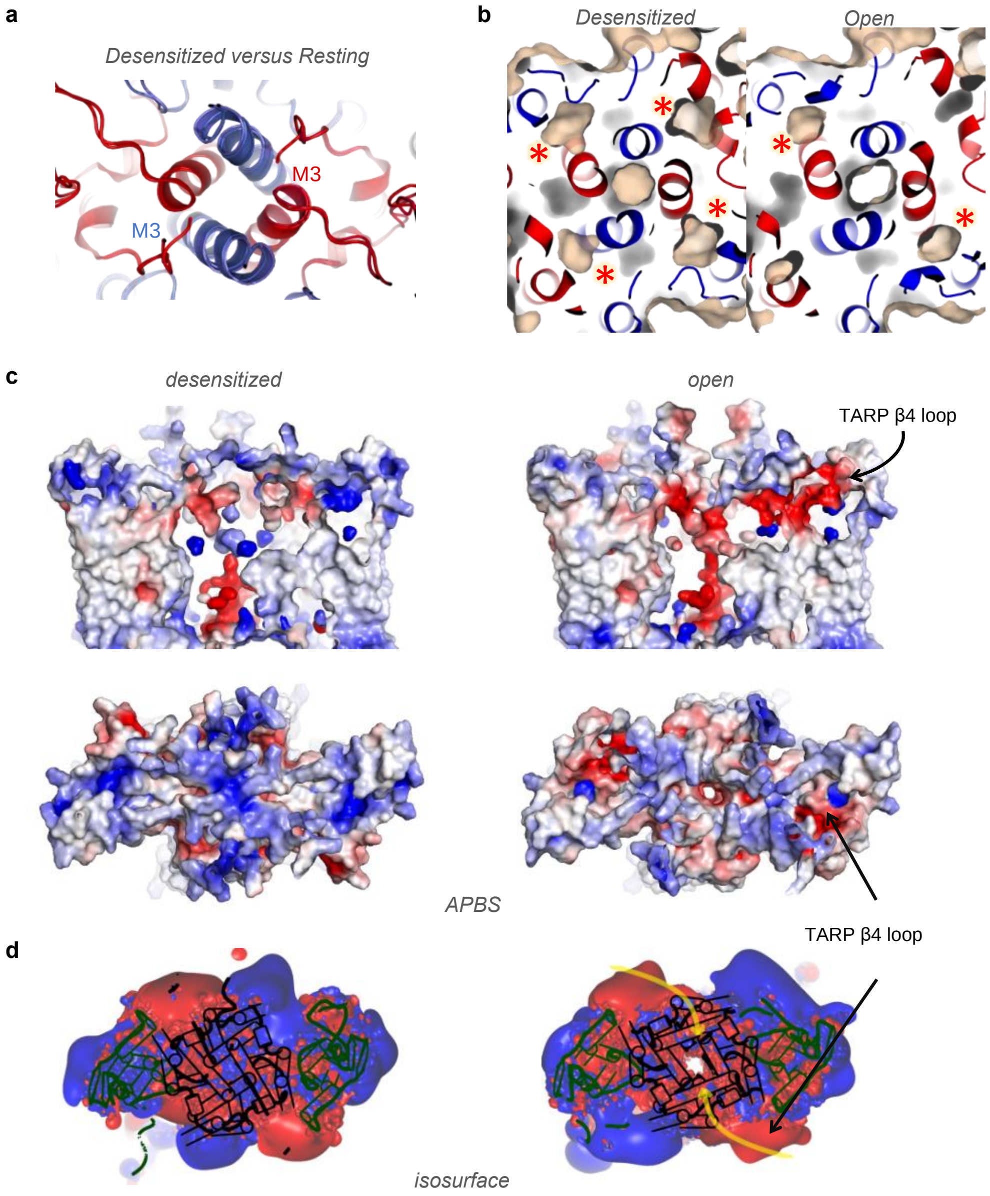

Supplementary Fig. 9: Analysis of electrostatics of the conduction path, pore entrance and TARP extracellular portion.

a, Superposition of open and desensitized states TMD M3 helices viewed from the extracellular region ("top view"), showing the high similarity between open and desensitized states in the transmembrane region. **b,** Cartoon representation of open and desensitized models viewed from the top, with the surface of the cavities represented in beige. The desensitized state contains 4 cavities for non-competitive antagonist binding, while only 2 sites are accessible in the open states (denoted with asterisks). **c,** Electrostatic surface of the TMD regions in open and desensitized states view from the side (parallel to the membrane) or top (perpendicular to the membrane) generated with APBS in PYMOL. **d,** The electrostatic isosurface shows the potential of 1 KbT/e (blue) and -1KbT/e (red). The calculation was performed using the APBS Electrostatics Plugin in Pymol with all default values. The core receptor (black) and TARP (green) is shown in the background; the yellow arrows indicate a negatively charged path leading from the TARP acidic loop towards the gate.

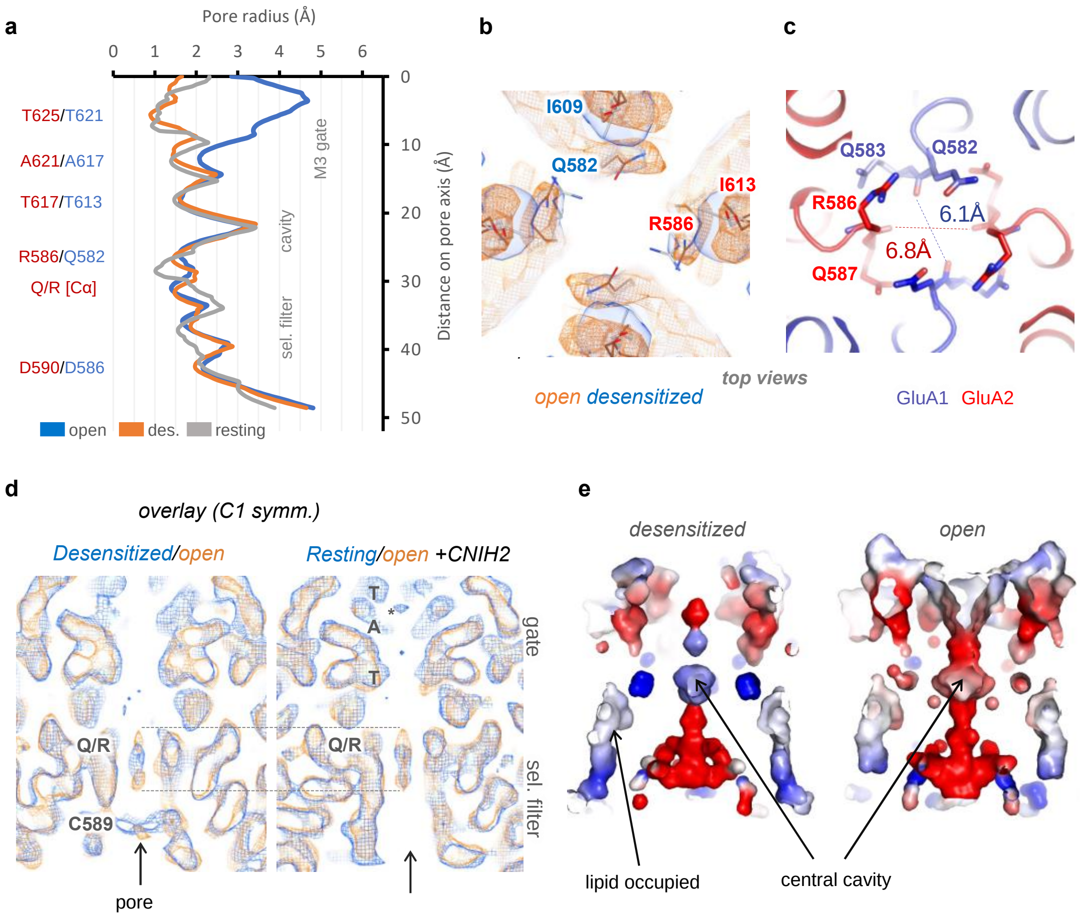

Supplementary Fig. 10: Analysis of conduction path and cation densities
a, Representation of the pore radius distances (X-axis) vs. the longitudinal distance (Y-axis) in open, desensitized and closed states, calculated with HOLE. The main residues lining the pore are indicated (GluA1 residues in blue and GluA2 residues in red). **b**, Comparison of the Q/R site densities in open (orange) and desensitized (blue) cryo-EM maps, with the fitted residues as lines **c**, Cartoon representation of the Q/R site of the open-state, with the main residues involved in the interaction represented as sticks and the distances between Arg586 and Gln582 carbonyl groups represented as dashed lines. **d,** Detail of the cryo-EM densities in open and desensitized states of GluA1/2/γ8 and GluA1/2/γ8/CNIH2, using COOT. The position of densities putatively corresponding to ions are separated with dashed lines. **e**, Comparison of the electrostatic surface in desensitized and open state models calculated with APBS. Open state shows a positively charged cavity which extends from the cytosolic side to the extracellular site, while the central cavity is negatively charged in the desensitized state. Range -10 kTe^{-1} to +10kTe^{-1}.

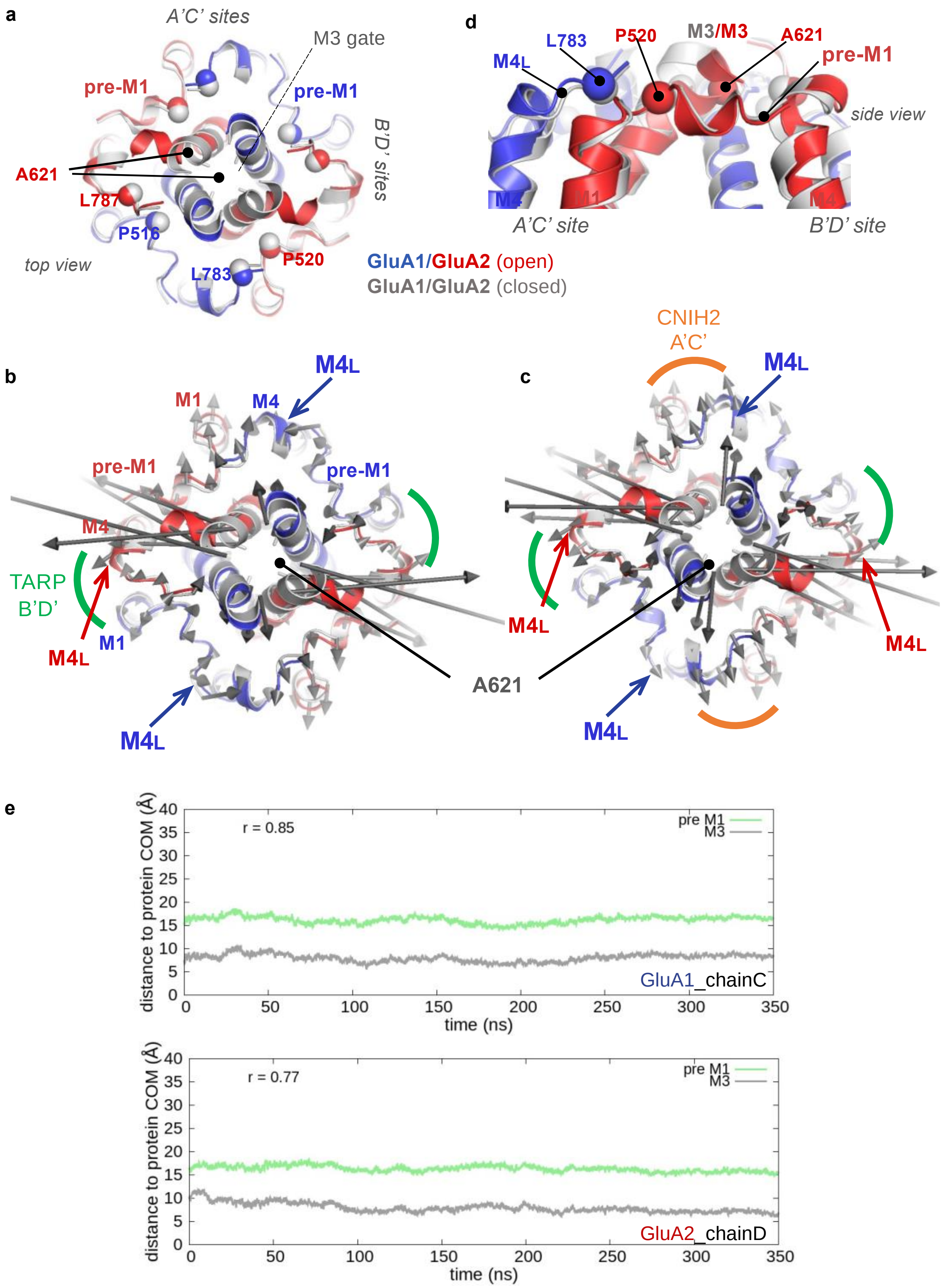

Supplementary Fig. 11: Structural and MD-simulation analysis of the gate surrounding region of TARP-only and TARP+CNIH2 AMPAR complex.

a, Top view onto the pre-M1/M4 gate-surrounding region, active (color) and desensitized (closed; grey), models are superimposed outlining opening of the M3 gate and expansion of marker atoms (spheres) of the gate-surround. Also shown are the A'C' and B'D' auxiliary subunit binding sites on the M1 and M4 helices. **b,c,** View as in panel (**a**), showing the TARPγ8-only (**b**) and the TARPγ8+CNIH2 (**c**) associated receptor. The vectors indicate motion accompanying the open-to-activated transition, which is larger in the presence of CNIH2. Auxiliary subunit binding sites are indicated (TARP, green; CNIH2, orange). **d,** Side view of the gating region showing how the gate

constricting Ala621 (GluA2) levels with the pre-M1/M4 linker fence. Dilation of this fence on activation is visible. **e, e,** Correlation of motion between a segment (N615/617-L-A-A-F-L) of the pore helix M3 and a segment (S512/516-F-L-D-P-L) of the pre M1 region (r, Pearson correlation coefficient). The motion was monitored across the simulations, as the distance of the center of mass of non-H atoms in these segments, from the pore center. Data shown is representative, for variations in chain C, GluA1 (top) and chain D, GluA2 (bottom) of the open state, Run6, over 350 ns simulations. For Chain C, pre M1: Mean=16.16, Std Dev: 0.79, M3: Mean= 7.96, Std Dev: 0.83, For Chain D, pre M1: Mean= 16.31, Std Dev: 0.67, M3: Mean= 7.90, Std Dev: 1.05. Source data are provided as a Source Data file.

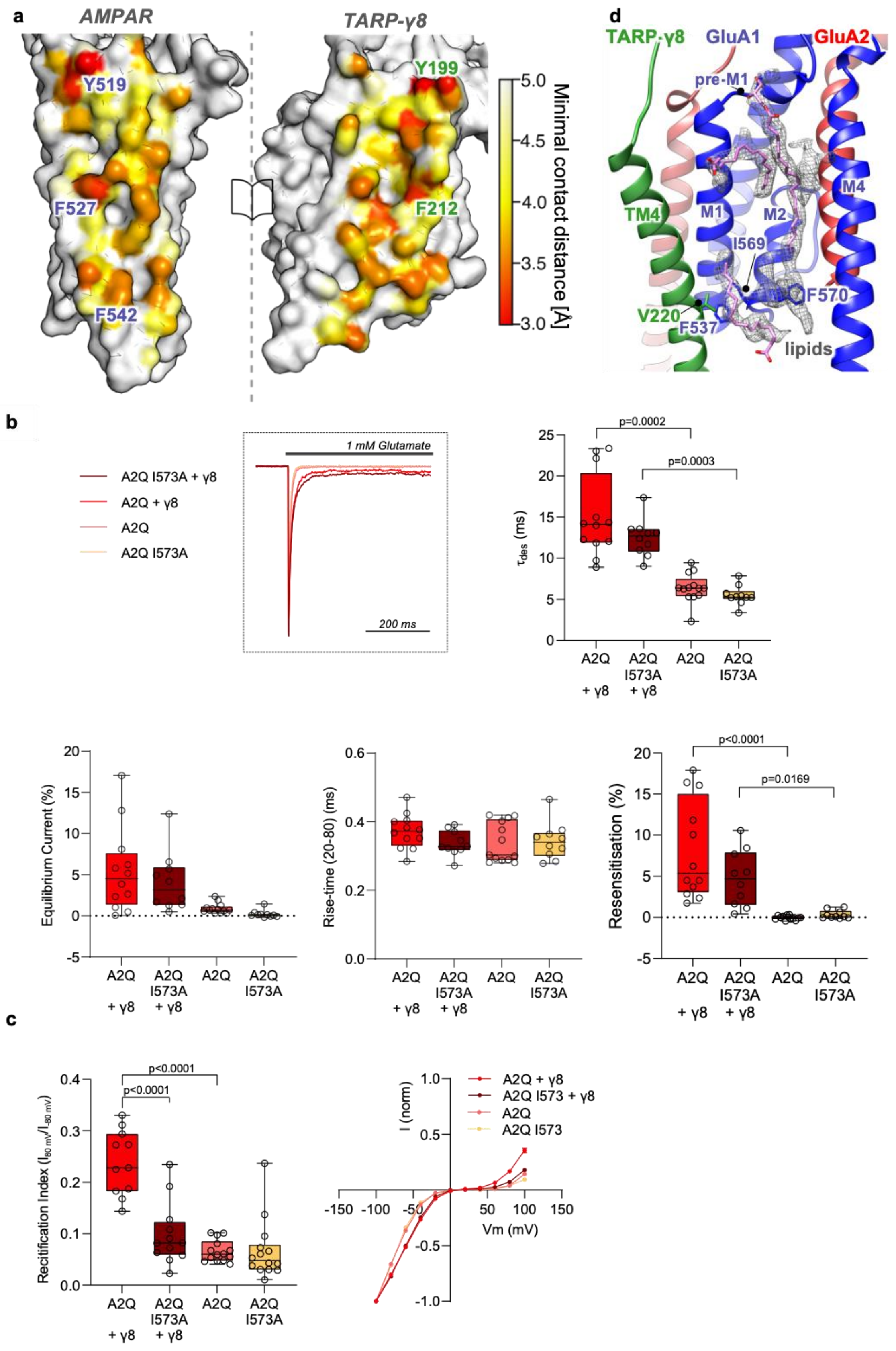

a
AMPAR
TARP-γ8
Y519
F527
F542
Y199
F212
Minimal contact distance [Å]
d
TARP-γ8
GluA1
GluA2
pre-M1
M1
M2
M4
TM4
I569
F570
V220
F537
lipids
b
A2Q I573A + γ8
A2Q + γ8
A2Q
A2Q I573A
1 mM Glutamate
200 ms
p=0.0002
p=0.0003
τdes (ms)
Equilibrium Current (%)
Rise-time (20-80) (ms)
p<0.0001
p=0.0169
Resensitisation (%)
c
p<0.0001
p<0.0001
Recitification Index (I80 mV/I-80 mV)
A2Q + γ8
A2Q I573A + γ8
A2Q
A2Q I573A
A2Q I573 + γ8
A2Q I573
I (norm)
Vm (mV)

Supplementary Fig. 12: TARP-γ8 interactions, lipid-binding sites and electrophysiological analysis of TARP-pore loop contacts.

a, 'Open book' representation of the GluA1 M1 ('AMPAR'; left) and TARP-γ8 (right) footprint. The closest atom-to-atom distance of all residues at the protein interface to any other residue of the opposing protein was calculated and color-coded. Measurements and visualization was performed in PYMOL. **b,** Glutamate-gated responses from GluA2Q associated with TARP γ8, with or without I573A mutation. Mutagenesis has no effect on rates of desensitization (Box (25 to 75 quartiles) and whisker (min to max values) plots are marked with the median value (line)) (weighted tau described as - Median (25, 75 quartiles). *A2Q + γ8*: *14.1* (11.9, 20.4) ms, *A2Q + I573A γ8*: 12.7 (10.8, 13.6) ms, *A2Q:* 6.4 (5.4, 7.5) ms, *A2Q I573A:* 5.3 (5.0, 6.0) ms, n = 10-13, two-tailed Kruskal-Wallis test, H(3) = 33.85, p<0.0001), equilibrium currents (*A2Q + γ8:* 4.52 (1.36, 7.62) %, *A2Q I573A + γ8:* 3.14 (1.33, 5.90) %, *A2Q:* 0.70 (0.48, 1.13) %, *A2Q I573A:* 0.10 (-0.05, 0.23) %, n=10-13 two-tailed Kruskal-Wallis test, H(3) = 24.27, p<0.0001), response rise-time (20-80 %, *A2Q + γ8:* 0.37 (0.33, 0.40) ms, *A2Q I573A + γ8:* 0.33 (0.32, 0.37) ms, *A2Q:* 0.30 (0.29, 0.41) ms, *A2Q I573A:* 0.34 (0.30, 0.37) ms, n=10-13, two-tailed Kruskal-Wallis test, H(3) = 3.52, p=0.32) or resensitization (*A2Q + γ8:* 5.35 (3.08, 15.00) %, *A2Q I573A + γ8:* 4.68 (1.52, 7.89) %, *A2Q:* -0.01 (-0.21, 0.19) %, *A2Q I573A:* 0.09 (-0.03, 0.78) %, n=10-13, two-tailed Kruskal-Wallis test, H(3) = 33.17, p<0.0001). Source data are provided as a Source Data file. **c,** *Left*: Current rectification index was significantly decreased by I573A mutagenesis specifically in γ8-associated receptors (*A2Q + γ8:* 0.23 (0.18, 0.29), n=11; *A2Q I573A + γ8:* 0.08 (0.06, 0.12), n=12; *A2Q:* 0.06 (0.05, 0.08), n=14; *A2Q I573A:* 0.05 (0.03, 0.06), n=14; two-tailed Welch's ANOVA test with Dunnett's T3 multiple comparisons test, F(3,21.39) = 23.76, p<0.0001), by reduction in outward currents. *Right*: Current-voltage (I/V) plot revealing a selective, TARP-γ8-dependent impact of GluA2 Ile573Ala on outward rectification (mean ± SEM, A2Q + *γ8*, n = 11; A2Q I573A + *γ8, n=12; A2Q, n=14; A2Q I573A, n=14*). Source data are provided as a Source Data file. **d,** Interaction region between TARP-γ8 TM4 (Val220) and GluA1 (M1, Phe537 and M2, Ile569, Phe570) involves lipid densities extending from the base of the M2 loop to preM1. These annular lipids also contact the M3 gating helix.

Supplementary Table 1: Data collection, processing and model statistics.

Data collection and processing	Open state GluA1/2_ γ8 (LBD/TMD)			Desensitized state GluA1/2_ γ8 (LBD/TMD)		
Microscope	Titan Krios			Titan Krios		
Location	Diamond Light Source			Diamond Light Source		
Magnification	81,000			81,000		
Voltage (kV)	300			300		
Detector	K3 with GIF			K3 with GIF		
Pixel size (Å)	1.06			1.06		
Electron exposure (e^-/ Å^2)	52			52		
Exposure length (s)	4			5		
Frame number	50			50		
Micrographs	9664			9664		
Final particle images (no.)	83,344			105,918		
	TMD/LBD	TMD	LBD	TMD/LBD	TMD	LBD
Map resolution (Å)	3.5	3.4	3.6	3.6	3.4	4.8
Symmetry imposed	C2	C2	C2	C2	C2	C2
FSC threshold	0.143	0.143	0.143	0.143	0.143	0.143
EMDB codes	13969	13970	13971	13972	13973	13973
Refinement						
Initial model used (PDB code)	6QKC /3TKD			6QKC/3TKD		
Model resolution (Å)	3.5			3.6		
FSC Threshold	0.5			0.5		
Map sharpening *B* factor (Å^2)	-111			-113		
Model composition						
Non-hydrogen atoms	15052			13626		
Protein residues	1962			1978		
Ligands	28			18		
Protein						
R.m.s deviations						
Bond lengths(Å)	0.024			0.014		
Bond angles (°)	1.114			1.767		
Ramachandran Plot						
Favoured (%)	90.9			99.6		
Allowed (%)	9.1			0.3		
Disallowed (%)	0			0.2		
Molprobity statistics						
Molprobity score	2.12			1.15		
Clash score	12.21			3.65		
Favored Rotamers (%)	99.1			97 %		
PBD codes	7QHB			7QHH		

Supplementary Table 2: Sequence of oligonucleotides.

Construct	Forward	Reverse
GluA1_γ8 I609A	GGTTCTTCACTTTGATCATCGCCTCCTCGT ACACAGCCAAC	GATGATCAAAGTGAA GAACCACCA
GluA2 I573A	GTGAATCAACTAATGAATTTGGGGCTTTT AATAGTCTCTGGTTTTCCTTGGGTGCC	CCCAAATTCATTAGTT GATTCACTACTTTGTG TTTC
GluA2_γ8 (γ2 β1)	GATTATTGGTTGTATACTAGGGCTGTTTGC AAGACCAAAAGTGTCAGTGAGAATGAAA CCAGCAAGAAGAACGAGGAAGTTATGAC TCACTCAGGACTGTGGCGGATTTG	AGCCCTAGTATACAAC CAATAATCTGTGG
GluA2_γ8 (γ2 ex2)	CCAATGCTGGGGAACCATCCAAGAGTGAC TCCAAGAAAAACCATTATTCCTACGGTTG GTCC	TGGTTCCCCAGCATTG GCGC
GluA2_γ8 glyco	CATATGTTATGATGAAGAAAAATCATACT ATGCTTGAAGGGAATGAGCGTTACGAG	ATGATTTTTCTTCATC ATAACATATGGAGATT CCAATATTG
GluA1 glyco	GTGATGCTTAAAAAGAATGCCACCCAGTT TGAGGGCAATGACCGCTATGAG	GGCATTCTTTTTAAGC ATCACGTAAGGATC
