# Supplementary material for: Mechanisms underlying TARP modulation of the GluA1/2-γ8 AMPA receptor
Source: Nat Commun. 2022 Feb 8;13:734. doi: 10.1038/s41467-022-28404-7 (PMC8826358; doi:10.1038/s41467-022-28404-7)
Supplement: Supplementary file 3 — Description of Additional Supplementary Files [file 41467_2022_28404_MOESM3_ESM.pdf]

Description of Additional Supplementary Files

File name: Supplementary Movie 1

Description: **Gating transitions of the GluA1/2 TARP-γ8 receptor**

Morph showing transition of the resting-state receptor to other states in the following order: activation – desensitization – rest; the movie starts with a side view, transits to top view and returns to side view). Color code: GluA1, blue; GluA2 red; γ8: green.

File name: Supplementary Movie 2

Description: **Open to desensitized transitions of the M3 gate and the selectivity filter entrance**

Cryo-EM maps of the GluA1/2 TARP-γ8 receptor (GluA1, blue; GluA2 red; γ8: green; lipids, grey). Top view zooming into M3 gate and selectivity filter at the level of the Q/R site, and transitioning between open and desensitized states.
