# Supplementary material for: Mechanisms underlying TARP modulation of the GluA1/2-γ8 AMPA receptor
Source: Nat Commun. 2022 Feb 8;13:734. doi: 10.1038/s41467-022-28404-7 (PMC8826358; doi:10.1038/s41467-022-28404-7)
Supplement: Supplementary file 6 — Reporting Summary [file 41467_2022_28404_MOESM6_ESM.pdf]

nature portfolio

Corresponding author(s): Ingo Greger

Last updated by author(s): Dec 17, 2021

Reporting Summary

Nature Portfolio wishes to improve the reproducibility of the work that we publish. This form provides structure for consistency and transparency in reporting. For further information on Nature Portfolio policies, see our Editorial Policies and the Editorial Policy Checklist.

Statistics

For all statistical analyses, confirm that the following items are present in the figure legend, table legend, main text, or Methods section.

n/a	Confirmed	
☐	☒	The exact sample size (*n*) for each experimental group/condition, given as a discrete number and unit of measurement
☐	☒	A statement on whether measurements were taken from distinct samples or whether the same sample was measured repeatedly
☐	☒	The statistical test(s) used AND whether they are one- or two-sided<br>*Only common tests should be described solely by name; describe more complex techniques in the Methods section.*
☒	☐	A description of all covariates tested
☐	☒	A description of any assumptions or corrections, such as tests of normality and adjustment for multiple comparisons
☐	☒	A full description of the statistical parameters including central tendency (e.g. means) or other basic estimates (e.g. regression coefficient) AND variation (e.g. standard deviation) or associated estimates of uncertainty (e.g. confidence intervals)
☐	☒	For null hypothesis testing, the test statistic (e.g. F, t, r) with confidence intervals, effect sizes, degrees of freedom and P value noted<br>*Give P values as exact values whenever suitable.*
☒	☐	For Bayesian analysis, information on the choice of priors and Markov chain Monte Carlo settings
☒	☐	For hierarchical and complex designs, identification of the appropriate level for tests and full reporting of outcomes
☐	☒	Estimates of effect sizes (e.g. Cohen's *d*, Pearson's *r*), indicating how they were calculated

Our web collection on statistics for biologists contains articles on many of the points above.

Software and code

Policy information about availability of computer code

Data collection	EPU, pClamp10, NAMD 3.0, GROMACS 2019.3
Data analysis	MotionCor2 v1.3.1, Gctf v1.18_b1_sm60_cu8.0, RELION 3.1, coot 0.9, PHENIX 1.18.2, REFMAC5, UCSF Chimera 1.14, Pymol 1.8.2.0, MolProbity v4.2, CCPEM v1.5, EMDA (https://www2.mrc-lmb.cam.ac.uk/groups/murshudov/content/emda/emda.html), pClamp10, GraphPad Prism 7.0e, ProDy 2.0, CHARMM-GUI v1.7, MODELLER 10.1, CONAN, VMD 1.9.4 a51

For manuscripts utilizing custom algorithms or software that are central to the research but not yet described in published literature, software must be made available to editors and reviewers. We strongly encourage code deposition in a community repository (e.g. GitHub). See the Nature Portfolio guidelines for submitting code & software for further information.

Data

Policy information about availability of data

All manuscripts must include a data availability statement. This statement should provide the following information, where applicable:

- Accession codes, unique identifiers, or web links for publicly available datasets
- A description of any restrictions on data availability
- For clinical datasets or third party data, please ensure that the statement adheres to our policy

Atomic coordinates have been deposited in the Protein Data Bank with accession codes 7QHB and 7QHH, and the cryo-EM density maps have been deposited in the Electron Microscopy Data Bank with accession codes EMD-13969, EMD-13970, EMD-13971, EMD-13972, EMD-13973, EMD-13974.

Field-specific reporting

Please select the one below that is the best fit for your research. If you are not sure, read the appropriate sections before making your selection.

☒ Life sciences ☐ Behavioural & social sciences ☐ Ecological, evolutionary & environmental sciences

For a reference copy of the document with all sections, see nature.com/documents/nr-reporting-summary-flat.pdf

Life sciences study design

All studies must disclose on these points even when the disclosure is negative.

Sample size	Cryo-EM sample sizes were determined by available electron microscopy time and the number of particles on electron microscopy grids. The sample size is sufficient to obtain a structure at the reported resolution, as assessed by Fourier shell correlation. Electrophysiology sample sizes were determined based on literature review, previous experience with data of this sort, and reproducibility of results across independent experiments. The authors have extensive previous experience with data of this type (Zhang, Nature 2021; Herguedas, Science 2019; Herguedas, Science 2016), therefore sample sizes were based on understanding of sample variabilities. The decision for sample sizes for MD simulations was made, based on the authors' previous experience with simulations of closely related systems, to obtain adequate conformational sampling for the purpose of this study.
Data exclusions	For cryo-EM, data were excluded using standard classification approaches in RELION to remove false picks and particle images without high resolution content. In electrophysiology experiments, data were excluded based on pre-established quality control criteria (rise time, holding current, and rectification index > 0.6 to maximize heteromeric receptor recordings). For MD simulations, all analyses were performed on the complete production runs; no data was excluded.
Replication	For cryo-EM, structures were determined from independent half datasets, which were compared to assess the resolution of the reconstruction. All electrophysiology data sets were pooled from at least two independent experiments and all results were successfully replicated. Replicate (n=2) MD simulations were performed for each system setup studied.
Randomization	For Cryo-EM, division of datasets into two random halves was done based on standard approach in RELION 3.1. Randomization is not relevant to electrophysiology experiments or MD simulations, as samples were not divided into experimental groups.
Blinding	Blinding was not applicable to cryo-EM or MD simulations, because this type of study does not use group allocation. Researchers were not blinded for the acquisition or analysis of electrophysiology data as it was not technically or practically feasible to do so. Experimenter independence was ensured by application of defined exclusion criteria as stated above.

Reporting for specific materials, systems and methods

We require information from authors about some types of materials, experimental systems and methods used in many studies. Here, indicate whether each material, system or method listed is relevant to your study. If you are not sure if a list item applies to your research, read the appropriate section before selecting a response.

Materials & experimental systems

n/a	Involved in the study	
☒	☐	Antibodies
☐	☒	Eukaryotic cell lines
☒	☐	Palaeontology and archaeology
☒	☐	Animals and other organisms
☒	☐	Human research participants
☒	☐	Clinical data
☒	☐	Dual use research of concern

Methods

n/a	Involved in the study	
☒	☐	ChIP-seq
☒	☐	Flow cytometry
☒	☐	MRI-based neuroimaging

Eukaryotic cell lines

Policy information about cell lines

Cell line source(s)	HEK293T cells were purchased from ATCC and HEK-Expi293F cells from ThermoFisher Scientific (Cat# A14527).
Authentication	No further authentication was performed for cell lines used in this study.
Mycoplasma contamination	No mycoplasma testing was performed specifically for this study, the HEK293T cell line had been tested negative in the past.
Commonly misidentified lines (See ICLAC register)	HEK cells are listed in the register; however, our HEK cell lines come from reliable source and are the only secondary cell type used in this study, which minimizes the risk of any cross-contamination.
